# Plant–environment interactions from the lens of plant stress, reproduction, and mutualisms

**DOI:** 10.1002/ajb2.1437

**Published:** 2020-02-14

**Authors:** Regina S. Baucom, Katy D. Heath, Sally M. Chambers

**Affiliations:** ^1^ Department of Ecology and Evolutionary Biology University of Michigan Ann Arbor MI 48109 USA; ^2^ Department of Plant Biology University of Illinois at Urbana‐Champaign Urbana IL 61801 USA; ^3^ Department of Botany Marie Selby Botanical Gardens Sarasota FL 34236 USA

Plants interact with a variety of abiotic and biotic environmental agents. They may rely on pollinators for reproduction, form beneficial mutualisms with microbial partners, or only grow and reach reproductive maturity in specific climatic conditions. Additionally, plants must survive a host of challenges from the environment, such as herbivore damage, low nutrient levels, or drought conditions. Plants deal with these factors in real time, without the ability to rapidly shift in geographic space as animals can. There are clearly multifaceted and intricate ways that plants must interact with environmental influences and inputs, and doing so is critical for both survival and reproduction. Thus, any efforts to understand plant evolution, growth, reproduction, distribution, and community structure include at some level the interactions plants have with the environment and the stressors they may encounter.

This cooperative special issue across journals features articles in the *American Journal of Botany* (*AJB*), *Applications in Plant Sciences* (*APPS*), and the *International Journal of Plant Sciences* (*IJPS*) that cover topics under the broad umbrella of plant–environment interactions. In *IJPS*, the plant–environment interaction is viewed from the paleobotanical (papers edited by Selena Smith, University of Michigan) and shape (edited by Dan Chitwood, Michigan State University) perspectives. The development of novel tools for studying plant–environment interactions is discussed in three publications featured in *APPS* (edited by Sally Chambers, Marie Selby Botanical Gardens). The website for each journal has links to the articles published as part of this cross‐journal special feature.

In this issue of *AJB*, the plant–environment interaction is considered from the lens of environmental stressors, mutualisms, and plant reproduction. In the stressful interactions section (edited by Regina Baucom, University of Michigan), the potential for adaptation and plasticity given components of the abiotic and biotic environment (light, temperature, minerals, water availability, elevational gradients, herbivores) are explored. In the living together section (edited by Katy Heath, University of Illinois), the evolutionary and ecological outcome of interactions between plants and their microbial partners (soil microbial communities, rhizobia, endophytic fungi) are considered, and finally, in the plant reproduction section (Jannice Friedman, Regina Baucom, Katy Heath, Sharon Kessler, and Georgiana May), interactions that influence mating system changes or pollination efficacy are examined. Below, we contextualize the diverse papers in this special feature that provide insight into myriad plant–environment interactions as well as descriptions of novel techniques developed to study these interactions.

## STRESSFUL INTERACTIONS

Plants can experience stress from a range of different and often multiplicative environmental factors. Contributions in this section consider such stressful interactions across broad scales: from an understanding of the genes, genetic pathways, and genome attributes that allow for adaptation to stressful agents, to an understanding of the process of adaptation to such factors, and finally to the ways in which plant community structure may be influenced by the stress of a changing climate. Three papers in this issue consider the genetics of adaptation to stress. Sanderson et al. (2020) provide insight into the genes underlying adaptation to freezing temperatures in natural *Arabidopsis* populations. They introduced a mutation that was originally found in the transcription factor *CBF2* in cold‐sensitive Italian lineages into cold‐tolerant Swedish backgrounds either through introgression or by using CRISPR‐Cas9. Interestingly, the experimental *CBF2* lines showed reduced freezing tolerance, and by examining differential regulation of the synthetic Swedish lines, the authors identified 10 additional genes involved in the freezing‐tolerance response. Another type of plant stress is the lack of adequate light for plant growth. Alameldin et al. (2020) examined genes involved in the block of greening response (BOG), which is the absence of greening in seedlings first exposed to far‐red light and then deprived of sucrose. This response allows for an investigation of the role of phytochromes and other regulatory genes involved in light perception. The authors expanded on previous work that identified the role of the mutant *phyA* in the BOG response by identifying a new mutant, *sig6,* which like *phyA,* allowed for greening after treatment that should have induced BOG. Finally, Wei et al. (2020) examined the types and patterns of trait changes that may occur when genomes double, which can allow for rapid evolutionary adaptation to new and stressful environments. Using synthetic *Fragaria* polyploids, they show that genome doubling alters traits such as stomatal length and density, as well as specific leaf area and vein density. Strikingly, these changes are similar to those of natural polyploid *Fragaria*, suggesting that genome doubling may play a significant role in adaptation in this genus.

A broad goal of evolutionary biologists is to determine the environmental factors that may impose stress and potentially act as agents of selection on plant populations. Two contributions to this issue consider environmental factors as agents of selection by performing either reciprocal transplants or long‐term analyses of selection in the wild. Popovic and Lowry (2020) used reciprocal transplants between inland and coastal lineages of *Mimulus guttatas* along with experimental exclosures that removed aboveground herbivores and salt spray. Strikingly, they found the fitness of inland lineages—which normally experience high rates of death in the coastal environment—to be completely rescued by exclosures at coastal sites, providing evidence that salt spray and/or herbivory were likely agents of selection. Murren et al. (2020) examined the potential for selection on root traits in natural and experimental populations of *Arabidopsis* over 4 years. Using multivariate analyses, they found that roots, which are critical for plant structural support and mineral and water uptake, exhibit differing patterns of selection temporally and spatially, with negative selection on root architecture traits in natural field populations and positive selection for total root length in experimental gardens.

A critical and ever‐encroaching form of plant stress comes from climate change, and three papers in this special issue examine plant adaptation or community changes in the context of a changing climate. MacTavish and Anderson (2020) examined the potential for local adaptation to nutrient and drought stress along an elevational gradient in *Boechera stricta*. They show that genetic lineages from low‐elevation, arid areas exhibit higher fecundity under extreme drought compared to families from more mesic, higher elevations, supporting local adaptation of lineages. They also show environmentally dependent fitness trade‐offs between growth and reproductive success: under high nutrient levels and extreme drought stress, there was a negative relationship between growth rate and the probability of reproduction, suggesting that such relationships might constrain adaptation to increasing drought and novel nutrient exposures. Similarly, Gremer et al. (2020) examined germination cues in *Streptanthus tortuosus* from populations also sampled along an elevational gradient. Differences in germination responses corresponded with both elevation and variability in seasonal temperature and precipitation across populations, and corresponded with germination phenology in the field. These two papers demonstrate that higher temperatures and decreased snowpack brought by a changing climate will alter important plant functional traits and that such changes will likely cascade to influence overall population persistence. Finally, Smithers et al. (2020) considered a biogeographic response to climate change. They examined the structure of alpine communities across elevational gradients in the White Mountains, California, United States, and found strong environmental sorting of alpine plant communities at broad scales, but that microclimatic and site‐specific, nonclimatic factors shape community turnover at fine scales. Such data are valuable in the context of climate change because they demonstrate that community–climate relationships are scale‐dependent and because current predictions of local alpine plant range shifts are limited by a lack of both topoclimatic and habitat information.

## LIVING TOGETHER

The interactions between mutualists and their hosts are complex, bi‐directional, and influenced by environmental factors. Contributions within this section considered both the idea that mutualists may alter host traits, and likewise, that host traits and the host environment may feed back to influence the quality of mutualism. For example, Christian et al. (2020) examined the role of secondary chemistry in mediating host affinity of the foliar endophytic fungi in the hosts *Psychotria* and *Theobroma* (cacao). They show that inoculation with fungal endophytes alters the secondary chemical profiles of host plants, which suggests either that plant secondary chemistry influences the composition of endophytes or that colonization by the endophytes themselves can influence changes in the host chemical landscape. While endophytic organisms may alter their hosts, the host genome can alter the type and quality of mutualisms that form. Using synthetic polyploids of *Medicago sativa*, Forrester and Ashman (2020) examined the effects of polyploidy on interactions with mutualistic microbes. They found that autotetraploids form larger nodules with larger zones of nitrogen fixation than diploids did when inoculated with two strains of *Sinorhizobium* rhizobia. Additionally, the environment experienced by the host can mitigate host–mutalist interactions. Heath et al. (2020) ask if variation in symbiont partner quality for their legume host plants is influenced by changing light availability. They show that light availability and symbiont inocula interact to influence plant responses to light, and moreover that variation in partner quality is more apparent in ambient light. Such results add to the growing recognition that intra‐ and interspecific microbial diversity plays an important role in mediating extended plant phenotypes.

Beyond endophytic relationships between plants and microbes, plants interact with the community of microbes found in the environment, and such interactions have high potential to influence plant adaptation. One way in which determining whether and how environmental microbial communities may mitigate plant adaptation is by determining whether such communities act as a selective agent on important plant traits. Chaney and Baucom (2020) show this to be the case by autoclaving soil to modify the soil microbial community. Doing so altered the pattern of selection on growth and flowering phenology in the common morning glory, *Ipomoea purpurea,* compared to plants growing in intact soils. These results indicate that the soil microbial community acts as an agent of selection on critical plant life history traits. Similarly, Batstone et al. (2020) examined the potential for different environments to influence selection and genetic variance in nodulation—a key trait reflecting legume investment in symbiosis—in *Medicago lupulina*. They found that mean and genetic variance for nodulation was greater in the greenhouse, yet selection was stronger in the field. Finally, of great interest is whether or not the plant microbiome may guard against the stress imposed by human modifications to the environment. O'Brien et al. (2020) investigated the impacts of common city stream contaminants—sodium chloride (salt) and benzotriazole (a common corrosion inhibitor)—on interactions between the duckweed *Lemna minor* and its microbiome. While they found that salt decreases both plant and microbial growth, benzotriazole provided a slight benefit to plant growth, but only when salt and microbes were absent. Importantly, they show that the presence of microbes did not buffer the negative influence of these stressors on their hosts, a finding contrary to the idea that microbial mutualisms might ameliorate plant stress.

## PLANT REPRODUCTION

Plant reproduction is a critical plant life history trait responsible for plant population persistence, and in this section, interactions between plants and their pollinators and the effect of environmental stressors on plant reproduction are highlighted. Lynn et al. (2020) examined the potential for pollinator‐mediated sexual selection on spines on the surface of *Taxacum* (dandelions) pollen. Interestingly, the authors show that pollen picked up by bumblebees exhibited a narrower subset of spine spacing phenotypes, consistent with stabilizing selection on pollen traits. In another contribution to this theme, Suni et al. (2020) investigated how water availability affects plant traits that influence pollination—flower size, nectar volume, and nectar sugar amount—and explored the role that local adaptation plays in responses to moisture availability. They found that drought led to smaller flowers and that in prolonged dry treatments, nectar volume and sugar remained higher in plants originally sourced from an arid region. These results suggest that plant investment in pollination mutualisms under environmental stress are adaptive and have the potential to change with climatic shifts. Another theme represented in this section of the special issue is the effect of plant injury on reproduction. In two contributed papers, Blake‐Mahmud and Struwe (2020a, b) examined how damage—specifically, defoliation—influences sex‐switching in *Acer pensylvanicum* and whether stored nonstructural carbohydrates influence sex expression. They show that severe damage such as full defoliation increases the odds an individual tree switches sex to female in the next year and that less‐severe physical trauma did not influence sex switching. They also show that female trees have higher sugar concentration than males and that males that changed sex expression to females had a higher sugar concentration the prior winter season compared to trees that remained male. Finally, Nihranz et al. (2020) also examine the effects of plant damage on reproduction, using a transgenerational approach in *Solanum carolinense* by looking at the consequences of both maternal plant herbivory and inbreeding on offspring reproduction. Maternal plants generated by inbreeding and by outbreeding were inflicted with weekly caterpillar herbivory, and the authors found influences of breeding type—offspring from inbred plants generally fared worse when it came to fitness—and an effect of herbivory, with offspring of herbivore‐damaged plants showing greater emergence, earlier flowering, and more flowers and seeds than offspring of undamaged plants.

## NOVEL TOOLS AND TECHNIQUES

The study of plant–environment interactions often involves a variety of field, manipulative, controlled environment, common garden, and computational approaches. Investigators may choose to test the importance of a single environmental factor in a controlled environment or manipulative experimental design to determine how plants respond to that factor in particular. Alternatively, a single factor can be manipulated in the field to study plant responses while other environmental factors are allowed to fluctuate naturally. In addition, computational approaches allow investigators to examine interactions across various trophic, spatial, and temporal scales, including paleoenvironments, future environments, or timelines that are difficult to work with in field or laboratory settings.

The three manuscripts contributed to *Applications in Plant Sciences* (APPS) in this cross‐journal special feature focus specifically on methods in the fields of plant responses to climate change, plant–pollinator interactions, and paleobotany and paleoclimates. Cranston et al. (2020) took a novel manipulative approach in a complicated field setting to examine the impacts of drought on large trees. This newly developed methodology is flexible and inexpensive; thus, it can be applied to a variety of study systems in areas that are difficult to access. Koptur et al. (2020) also took a manipulative approach to address a plant–pollinator question in a common garden system. Using different widths of fishing line, the authors demonstrate that they can identify the insects that successfully pollinate members of the Apocynaceae family. This plant family is riddled with complex floral morphologies and pollination mechanisms; thus, the methods developed here may be useful in other complex floral systems. Examining plant interactions with the environment under historical and future conditions is possible via the use of computational approaches. In their contribution, Harbert and Baryiames (2020) present a new R package that uses previously developed cRacle analyses to estimate historical climatic conditions from plant community records. When implementing this package, users are able to access data from popular repositories, aggregate said data and generate models which estimate climate based on documented vegetation.

Each of these three contributions to the cross‐journal special feature are generally inexpensive and easy to access, and as such we believe that these novel approaches will assist researchers as they investigate plant–environment interactions across a variety of subdisciplines.

## CONCLUSIONS

Plants interact with their environment in diverse ways, and the outcome of such interactions may influence trait evolution, population persistence, and overall community structure. The broad sample of publications in this special issue represent a snapshot of the various ways in which plants interact with their environment and the outcomes of such interactions. The contributions reflect the broad and diverse ways in which researchers think about and study plants in the context of their environment and highlight the interdisciplinary and diverse nature of such undertakings.
